# Validity of the Clock Drawing Test in predicting reports of driving problems in the elderly

**DOI:** 10.1186/1471-2318-4-10

**Published:** 2004-10-29

**Authors:** Nathan M Diegelman, Alan D Gilbertson, Jeffrey L Moore, Evangelia Banou, Michael R Meager

**Affiliations:** 1 Department of Psychiatry & Behavioral Sciences, Akron General Medical Center, Akron, OH 44307, USA; 2 Department of Psychology, Kent State University, Kent, OH 44242, USA; 3 Department of Psychology, Mount Union College, Alliance, OH 44601, USA

## Abstract

**Background:**

This study examined the use of the Folstein Mini Mental Status Exam (MMSE) and the Clock Drawing Test (CDT) in predicting retrospective reports of driving problems among the elderly. The utility of existing scoring systems for the CDT was also examined.

**Methods:**

Archival chart records of 325 patients of a geriatric outpatient clinic were reviewed, of which 162 had CDT results (including original clock drawings). *T*-test, correlation, and regression procedures were used to analyze the data.

**Results:**

Both CDT and MMSE scores were significantly worse among non-drivers than individuals who were currently or recently driving. Among current or recent drivers, scores on both instruments correlated significantly with the total number of reported accidents or near misses, although the magnitude of the respective correlations was small. Only MMSE scores, however, significantly predicted whether or not any accidents or near misses were reported at all. Neither MMSE nor CDT scores predicted unique variance in the regressions.

**Conclusions:**

The overall results suggest that both the MMSE and CDT have limited utility as potential indicators of driving problems in the elderly. The demonstrated predictive power for these instruments appears to be redundant, such that both appear to assess general cognitive function versus more specific abilities. Furthermore, the lack of robust prediction suggests that neither are sufficient to serve as stand-alone instruments on which to solely base decisions of driving capacity. Rather, individuals who evidence impairment should be provided a more thorough and comprehensive assessment than can be obtained through screening tools.

## Background

Assessment of cognitive function pertaining to capacity for safe and independent living among elderly patients is a central responsibility of many geriatric medical clinics and service agencies. Specific concerns pertaining to judgments of driving capacity are also befalling upon the medical profession in primary care settings [1]. To aid in this task, a number of brief assessment screens are often employed to identify cognitive problems that may be indicative of a range of pragmatic concerns, including driving capacity [2]. Specifically, results on assessment instruments purported to assess attention, reaction time, and visuospatial abilities are often used to inform clinical judgment about driving capacity in such settings. Two such screening instruments typically used to gauge general cognitive function, and inform questions pertaining to driving capacity specifically, are the Folstein Mini Mental Status Exam (MMSE) [3] and the Clock Drawing Test (CDT).

The MMSE is a widely used cognitive screening tool, due to its brevity, ease of administration, and relative breadth [4]. Numerous studies over the past 40 years have supported its utility as a valid and reliable indicator of general cognitive function [5]. The MMSE consists of 30 items comprising subscales assessing orientation, word registration, attention (via a serial sevens or spelling task), word recall, and language. Additionally, a figure copy exercise is included to examine visuospatial abilities. The CDT is hypothesized to assess more specific aspects of planning, organization and visuospatial skill. Directions for completing the CDT involve asking a patient to draw the face of a clock, including the numbers, and then to place the hands to designate a certain time, such as "ten minutes after eleven." Although different scoring templates for the CDT exist, most often code for features such relative size, spacing and placement of numbers or hands, disorganization, perseveration, completeness, and other potential errors that are hypothesized to indicate cognitive impairment [6-8].

In addition to the MMSE, results on the CDT are often used in clinical settings to inform clinical impressions pertaining to whether or not patients are impaired to such an extent that they should not be driving [9]. Although empirical reviews note that performance on the CDT should only be examined in conjunction with other assessments in this regard, anecdotal evidence also suggests that the CDT is often used as a stand-alone instrument to inform judgments of driving capacity, in both medical and non-medical settings.

Despite this apparent widespread use, there appears to be a dearth of research addressing the validity of the CDT for detecting driving impairment. Although a small number of studies exist that suggest CDT scores may relate to driving problems, the size of this literature base coupled with methodological concerns indicate a need for further research. For example, one study [10] examined the effectiveness of the CDT and MMSE, in addition to the Trail Making Test, Part A [11] and a visual acuity test, in predicting driving ability as judged by a driving instructor after participants completed a road test. A discriminant function analysis indicated that the set of test scores and participant age correctly identified 80% of drivers judged to be impaired, and 85% of drivers judged not to be impaired, according to driving instructor assessments. The authors reported that the discriminant model did not include the MMSE, however, because it did not add significant discriminatory power. The authors then suggested that the overall battery may be useful as a screening instrument in primary health care settings for detecting potential problems in driving that would warrant further examination. Separate univariate data on the predictive power for each of the separate instruments, however, was not provided. Additionally, the authors incorporated a 4-point scoring system for the CDT that was created for the study and differs from scoring systems used in other studies. Furthermore, given that only the component instruments are typically used in practice as opposed to the more extensive batteries advocated, the unique predictive power of the CDT warrants further investigation.

Additional evidence for the potential utility of the CDT in predicting driving behaviors is provided in an examination of neurophysiologic phenomena related to caregiver reports of driving impairment in 79 individuals with Alzheimer's disease [12]. Single photon emission computerized tomography was incorporated to examine brain function. Additionally, scores on the MMSE, CDT, and caregiver ratings of driving ability were analyzed. CDT scoring was based upon a 5-point system that was constructed for the study. Results indicated that MMSE scores did not significantly differ between individuals based upon driving ability, but that CDT scores were predictive of driving impairment based upon level of impairment and whether participants were instructed to simply copy an existing clock, or construct their own according to specific directions. Furthermore, imaging also indicated that level of driving impairment related positively to changes in cortical function. These authors hypothesized that cognitive tests assessing visuospatial abilities and executive function may thus show greater discriminative power between driving impaired and non-impaired subjects than MMSE scores, which may be impacted to a greater extent by other non-relevant verbal tasks. The validity of the scoring system constructed for the CDT in comparison to other scoring systems, however, was not further explored.

A pilot study examining the comparability of simulated driving tests in predicting actual driving problems also suggested that CDT scores may be significant predictors [3]. A small sample of nine older adults was incorporated, four of whom were classified as cognitively impaired based in part on abnormal CDT and MMSE scores. It was found that simulated driving tasks correlated moderately with actual driving problems across the groups. No data was provided, however, on the extent to which the CDT or MMSE uniquely predicted impairment.

Given the typically low rate of follow-up for patients referred for more formal driving assessments, it would be beneficial to further investigate the relations between scores on the CDT and reports of actual driving problems. Furthermore, the predictive power of the CDT alone and in conjunction with other assessment tools in predicting reported driving problems has yet to be fully assessed. Additionally, previous studies examining CDT scores and driving behaviors have employed markedly small sample sizes, warranting future research with greater numbers of participants. Finally, previous studies differ in terms of what, if any, scoring systems were used to score the clock drawings. Thus, further investigation of the comparability of different scoring systems is needed.

To address these concerns, this study explored the relations of patient scores on the CDT and MMSE to patient or family reports of driving problems. In so doing, the utility and comparability of three scoring systems for the CDT that are commonly used by researchers and practitioners, namely the Shulman et al. [6], Sunderland et al. [7], and Wolf-Klein et al. [8]systems, were also examined. Specifically, the Shulman system incorporates a 1–6 rating scale, where higher scores indicate higher levels of impairment. Conversely, scores on the Wolf-Klein and Sunderland systems range from 1–10, with lower scores indicating greater levels of impairment. Although specific criteria differ, each system codes for elements pertaining to spacing, organization, and comprehension of the task, among other criteria.

Exploratory analyses also were conducted to examine the predictive utility of the CDT and MMSE in predicting whether driving problems, namely accidents or near misses, were reported. Further analyses examined whether linear relationships existed between CDT and MMSE scores and the reported number of such incidents. Finally, regression tests examined whether the CDT and MMSE uniquely predicted the number of reported incidents.

## Methods

IRB approval was obtained for the study, and data was collected from archival records of patients seen over a 10-year period at a geriatric assessment center of a general teaching hospital in the Midwest. The center operated as a full-service outpatient clinic, where new patient assessments included a full medical and psychosocial history. This history included patients' and collateral others' reports of driving behaviors within the past year, including whether patients were currently driving or had recently stopped driving within the past year, and number of driving accidents or near misses. The data was often collected during the initial intake assessment, when both the patient and available family members or caregivers were interviewed by a geriatrician, social worker, and/or a nurse specialist. In addition, the MMSE and CDT were typically administered to patients to assess cognitive functioning.

Chart records did not clarify whether the reported driving problems were acknowledged by the patient or caregiver, nor the extent to which any discrepancies existed, but rather only reported the number of incidents. The content of the incidents was also not always documented, but examples that were provided typically included crashes or minor accidents for which the patients were at fault. Nevertheless, despite the subjectivity inherent to such reports, it was the intent of these authors to remain true to the figures documented in the patient charts. Indeed, given that medical professionals typically must rely to some extent on subjective reports of patients or caregivers during intake evaluations to inform initial judgments about patient safety, it was decided that incorporation of such data in the present study would nonetheless be useful.

Data was obtained from charts of 325 patients, including 162 original clock drawings that were scored according to the systems provided by Shulman et al. [6], Sunderland et al. [7], and Wolf-Klein et al. [8]. Two advanced students in psychology were trained in each of the three scoring systems, and subsequently scored the clocks independently of each other and blinded to information about driving. MMSE scores, driving status, and reports of driving problems were also coded for subsequent analyses. The initial sample consisted of 81 men and 242 women (gender data was unavailable for 2 individuals). Of these, 287 (88.3%) were Caucasian, 34 (10.5%) were African American, and one individual was Asian American. Ethnicity data was not available for the other three individuals. The mean patient age was 79.75 (*SD *= 6.67), and ranging from 58 to 99 years of age. As is typical of many outpatient geriatric populations, there was a range in type and severity of presenting concerns, with some patients reporting relatively few problems and others evidencing diagnoses of vascular dementia, Alzheimer's disease, or depression in addition to other health concerns. Of these, concerns due to cognitive function predominated; approximately 60% of the patient sample was referred to the clinic for evaluation of memory loss, cognitive decline, or dementia. MMSE data was available for 311 patients; of these, 159 also had CDT data sufficient for analysis. Of the 162 charts that had CDT data, only 3 did not also have MMSE data.

## Results and discussion

The raters' corresponding CDT scores for each scoring system correlated above 0.70, suggesting adequate inter-rater correspondence. The corresponding scores for each scoring system were then averaged to create three composite scores for each clock drawing, one for each scoring system. Descriptive data pertaining to scores for the overall sample on the MMSE and CDT is provided in Table 1.

**Table 1:** Descriptive statistics for overall sample scores on cognitive measures

Test	*N*	Mean	*SD*	Range
MMSE	311	21.51	6.15	0–30
CDT (Shulman Score)	162	3.87	1.25	1–6
CDT (Wolf-Klein Score)	162	6.58	2.10	1–10
CDT (Sunderland Score)	162	6.35	2.49	1–10

Initial exploratory *t*-tests were conducted to examine whether CDT scores and MMSE scores differed between individuals who had been currently or recently driving, versus those who had not been reported to be driving for a more extensive time period. In each case, current and recent drivers evidenced better scores on all of the cognitive measures than individuals who had not been driving. Results for these analyses are provided in Table 2.

**Table 2:** Mean differences in CDT and MMSE scores based on driving status

Variable	*N*	Mean	*SD*	*t*	*df*
MMSE Score					
Currently or Recently Driving	114	24.32	4.87	6.41**	305
Not Currently driving	193	19.98	6.18		
Shulman Score					
Currently or Recently Driving	61	3.39	1.28	-3.94**	157
Not Currently driving	98	4.16	1.15		
Wolf-Klein Score					
Currently or Recently Driving	61	7.26	1.83	3.29**	157
Not Currently driving	98	6.18	2.13		
Sunderland Score					
Currently or Recently Driving	61	7.18	2.28	3.28**	157
Not Currently driving	98	5.89	2.49		

Note. **p < .01.

Further analyses examined whether CDT or MMSE scores predicted the presence of reported driving problems among individuals who had been current or recent drivers. Patients who had not been driving for a longer period of time were excluded from the analyses, since no driving problems would have been reported as a function of not driving. Specifically, *t*-tests were incorporated to examine whether CDT and MMSE scores differed among individuals for whom driving problems had been reported, versus those with none. Drivers with reported problems evidenced significantly lower MMSE scores, but no significant differences were obtained for CDT scores. Nevertheless, the trends for the overall mean differences on CDT scores, although small, were in the same direction as the findings for the MMSE. Specifically, in every case the CDT scores for each scoring system were worse for drivers with reported problems than those with none. Overall, these results suggest that the presence of driving problems may have been reflective of greater levels of cognitive impairment, although the overall differences reflected in CDT scores were nonetheless very small in magnitude. These results are detailed in Table 3.

**Table 3:** Mean differences in CDT and MMSE scores based on presence of reported driving problems among current or recent crivers

Variable	*N*	Mean	*SD*	*t*	*df*
MMSE Score					
Did Report Problems	51	23.08	6.03	-2.44*	112
Did Not Report Problems	63	25.32	3.41		
Shulman Score					
Did Report Problems	27	3.57	1.35	1.03	59
Did Not Report Problems	34	3.23	1.22		
Wolf-Klein Score					
Did Report Problems	27	7.09	2.25	-.64	59
Did Not Report Problems	34	7.40	1.43		
Sunderland Score					
Did Report Problems	27	6.72	2.64	-1.41	59
Did Not Report Problems	34	7.54	1.91		

Note. *p < .05.

Next, the linear relations for both CDT and MMSE scores in predicted the number of reported problem incidents were examined. Specifically, correlation coefficients were calculated separately for number of reported accidents or near misses, and scores on the CDT and MMSE. Patients who had not been currently or recently driving were excluded from the analysis, since no problems would have been reported if they had not been driving. The number of reported incidents correlated significantly and positively with the level of cognitive impairment as measured by MMSE and CDT scores. Additionally, each of the CDT scoring systems appeared to evidence similar predictive utility, as they correlated highly (above 0.80). Means, standard deviations, and correlations for these variables are provided in Table 4. Given that not all patient charts necessarily contained all of the requisite MMSE and CDT data, cases that were missing data were excluded from some of the cells. Thus, the *n *of the resultant cases is included for each cell.

**Table 4:** Means, standard deviations, and correlations for cognitive measures and reported number of problems among current or recent drivers

Variable	*N*	*M*	*SD*	1	2	3	4
1. MMSE Score	114	24.32	4.87				
2. Shulman Score	61	3.39	1.28	-.45** (59)			
3. Wolf-Klein Score	61	7.26	1.83	.50** (59)	-.80** (61)		
4. Sunderland Score	61	7.15	2.30	.58** (59)	-.82** (61)	.83** (61)	
5. Reported Number of Driving Problems	116	.62	.90	-.27** (110)	.23* (57)	-.24* (57)	-.27* (57)

Note. ** p < .01, *p < .05. The *N *for each cell is provided in parentheses. Total reported number of driving problems ranged from 0–4 for each patient.

Finally, hierarchical regression analyses examined whether MMSE or CDT scores uniquely predicted number of reported accidents or near misses. The non-significant R-squared change term in the second step of each regression indicates that neither the MMSE nor set of CDT scores predicted significant incremental variance. Thus, it appears that the variance in reported accidents or near misses predicted by the MMSE and CDT was redundant. Regression results are provided in Table 5.

**Table 5:** Hierarchical regression analyses predicting number of reported driving problems from CDT and MMSE scores among current or recent drivers (*N *= 54)

Regression Criterion and Steps	*R*	*R^2^*	*F*	*df*	*R^2^*_change_	*F*_change_
Reported Number of Accidents or Near Misses						
Step 1: CDT Scores	.25	.06	1.17	3,51	.06	1.17
Step 2: MMSE Score	.31	.10	1.37	1,50	.04	1.92

Step 1: MMSE Score	.30	.09	5.19*	1,53	.09	5.19*
Step 2: CDT Scores	.31	.10	1.37	3,50	.01	.18

Note. *p < .05

## Conclusions

The results of this study suggest that both the MMSE and the CDT appear to have only limited utility in predicting retrospective reports of driving problems among elderly drivers. The finding that MMSE and CDT scores were worse among patients who had not been currently or recently driving may be due to a number of factors, including the possibility that some individuals may have never driven at all before. Nevertheless, it appears likely that many of these individuals probably had been driving in the past, but may have since stopped due to problems related to cognitive impairment. This assertion is supported by the finding that individuals who had been currently or recently driving at the time of the assessment, and who had lower MMSE scores, were more likely to have had reports of accidents or near misses. Although similar mean tests with the CDT were not significant, it is notable that the direction of the obtained differences for each scoring system of the CDT was consistent with the findings of the MMSE. Furthermore, the relatively modest *n*-sizes within each cell may have limited statistical power.

More robust findings were obtained, however, for the correlations examining number of reported accidents or near misses to CDT and MMSE scores. Among individuals who had been currently or recently driving at the time of assessment, greater levels of cognitive impairment as evidenced by MMSE and CDT scores also predicted greater numbers of reported accidents or near misses. This finding held regardless of which CDT scoring system was incorporated, suggesting that each may have equal utility.

Finally, the results of the regression analyses appear to indicate that the predictive power of the CDT and MMSE are somewhat redundant, since neither added significant incremental variance to prediction. Although it is possible that the regressions may have had limited power to detect significant incremental differences due to the relatively small sample sizes, in each case the increment to R-squared was small nonetheless. Thus, it appears that both the MMSE and CDT served as gross assessments of general cognitive function, versus more specific cognitive capacities, in predicting reported numbers of accidents or near misses.

Although the current results appear to suggest limited predictive utility for the MMSE and CDT in predicting driving problems, an additional cautionary note is in order. The significant predictive power for each instrument as demonstrated by the magnitudes of the correlation coefficients was nevertheless small. Furthermore, significant predictive utility was not obtained for every test in the current research. Additionally, the use of a retrospective design does not necessarily allow for definitive conclusions about predicting instances of future driving problems. Thus, although poor CDT or MMSE scores appear to indicate greater potential for driving problems, the current data do not support the use of the CDT or MMSE alone in making definitive decisions pertaining to driving competence. Rather, the empirical findings of the current research appear to best support the use of the CDT and MMSE solely as their originally intended purpose as screening tools. Thus, scores evidencing impairment on either of these instruments may indicate a need for driver caution, followed by more comprehensive and extensive assessment of driving capacity on which to base decisions regarding safety. As such, the role and utility of these instruments in predicting driving problems may be more fully understood through future research that incorporates a prospective design, along with a more comprehensive assessment of specific and relevant cognitive skills (like psychomotor speed or executive function) and objective assessment of driving abilities (such as can be obtained through simulated or practice driving situations).

## Competing interests

The author(s) declare that they have no competing interests.

## Authors' contributions

ND, AG, and JM conceived of the study purpose and design. ND, EB, and MM collected, entered, and analyzed the data. ND conducted the literature review and critique, and drafted the manuscript. AG, JM, EB, and MM provided comments on the manuscript. All authors read and approved the final manuscript.

## Pre-publication history

The pre-publication history for this paper can be accessed here:


